# Associations of Maternal rs1801131 Genotype in *MTHFR* and Serum Folate and Vitamin B_12_ with Gestational Diabetes Mellitus in Chinese Pregnant Women

**DOI:** 10.3390/nu14061169

**Published:** 2022-03-10

**Authors:** Shuying Li, Xiubiao Tian, Yiyun Wang, Xumei Zhang, Liwen Zhang, Chen Li, Jing Li, Chunhua Wang, Huihuan Liu, Juan Liu, Hongjuan Liu, Xueli Yang, Weiqin Li, Junhong Leng, Xilin Yang, Naijun Tang, Qiang Zhang

**Affiliations:** 1 Department of Endocrinology, Tianjin Xiqing Hospital, Tianjin 300380, China; lsy_tj2006@163.com (S.L.); 15022754681@163.com (X.T.); 2 Department of Occupational and Environmental Health, School of Public Health, Tianjin Medical University, Tianjin 300070, China; wyy2168390@163.com (Y.W.); zhangliwen@tmu.edu.cn (L.Z.); lichen@tmu.edu.cn (C.L.); wangchunhua@tmu.edu.cn (C.W.); yangxueli@tmu.edu.cn (X.Y.); tangnaijun@tmu.edu.cn (N.T.); 3 Tianjin Key Laboratory of Environment, Nutrition and Public Health, School of Public Health, Tianjin Medical University, Tianjin 300070, China; zhangxumei@tmu.edu.cn; 4 Department of Nutrition and Food Science, School of Public Health, Tianjin Medical University, Tianjin 300070, China; 5 Department of Epidemiology and Biostatistics, School of Public Health, Tianjin Medical University, Tianjin 300070, China; lijing_epi@tmu.edu.cn (J.L.); yangxilin@tmu.edu.cn (X.Y.); 6 Beichen District Women’s and Children’s Health Center, Tianjin 300499, China; 18522022122@163.com; 7 Department of Biomedical Information and Library, Tianjin Medical University, Tianjin 300070, China; libliujuan@tmu.edu.cn; 8 Department of Obstetrics and Gynecology, Tianjin Xiqing Hospital, Tianjin 300380, China; juanliuhong@163.com; 9 Project Office, Tianjin Women and Children’s Health Center, Tianjin 300070, China; liweiqin007@163.com (W.L.); ljhlzqljhlzq@163.com (J.L.)

**Keywords:** MTHFR, folate, vitamin B_12_, one-carbon metabolism, gestational diabetes mellitus

## Abstract

Circumstantial evidence links one-carbon metabolism (OCM) related nutrients, such as folate and vitamin B_12_, with gestational diabetes mellitus (GDM). However, few studies have evaluated the combined effects of these nutrients with OCM related gene polymorphisms on GDM. This study investigated whether OCM related genetic variants modified the associations of folate and B_12_ with GDM. Logistic regression was used to estimate odds ratios (ORs) for OCM related nutrients and single nucleotide polymorphisms (SNPs) in genes encoding main OCM related enzymes (*MTHFR*, *MTR*, and *MTRR*) on GDM. Higher folate concentrations were associated with increased GDM risk (OR: 1.59; 95% CI: 1.22, 2.13). However, higher B_12_ concentrations were associated with reduced GDM risk (OR: 0.76; 95% CI: 0.65, 0.92). Pregnancies with *MTHFR* rs1801131 G alleles had a significantly lower risk of GDM than pregnancies with T alleles (OR: 0.65; 95% CI: 0.47, 0.91) under the dominant model. The genotype-stratified analysis revealed the association between folate and GDM (OR: 1.66, 95% CI: 1.20, 2.30) or B_12_ and GDM (OR: 0.80, 95% CI: 0.65, 0.98) was more evident in pregnancies with TT genotype. Higher folate and lower B_12_ are associated with GDM. Pregnancies with *MTHFR* rs1801131 TT genotype are more susceptible to OCM nutrient-related GDM.

## 1. Introduction

Gestational diabetes mellitus (GDM), defined as glucose intolerance with onset or first recognition during pregnancy, is currently the most common medical complication. The prevalence of GDM varies considerably among countries, ranging from 1.8% to 31% [1]. Aside from its short-term adverse consequences, GDM brings long-term negative effects for both mothers and their offspring, notably, a higher risk of developing type 2 diabetes mellitus for mothers after pregnancy [2]. Therefore, it highlights the importance of identifying risk factors related to GDM and developing effective prevention strategies before diagnosing GDM. Since the lifestyle intervention on GDM is a limited success [3,4], it is essential to identify other risk factors to prevent GDM.

Folate and vitamin B_12_ (B_12_) are essential micronutrients required during pregnancy. They are involved in DNA methylation and biosynthesis of nucleic acids and proteins required for cellular replication and fetal growth [5]. They are metabolically interlinked in the one-carbon metabolism (OCM) pathway. In this pathway, a methyl group is transferred from serine to tetrahydrofolate (THF) to form 5, 10-methylene-THF, which is subsequently reduced to 5-methyl-THF via methylenetetrahydrofolate reductase (*MTHFR*). The methyl group of 5-methyl-THF is transferred to homocysteine (Hcy) by the B_12_-dependent methionine synthase (*MTR*) and methionine synthase reductase (*MTRR*), generating methionine and THF [6]. Hcy is a sensitive marker of folate and B_12_ deficiency since B_12_ is a cofactor for the folate-dependent remethylation of Hcy to methionine.

Recently, the role of OCM related nutrients on the development of GDM has emerged as a field of public interest. Folic acid supplements are widely recommended for women of reproductive age before and during early pregnancy to prevent neural tube defects [7]. With increased folate intake, concerns regarding the potential health risk of folate excess have been raised in recent years. Several prospective cohort studies have shown that both folate supplementation and blood folate levels are associated with an increased risk of GDM [8,9,10]. However, another prospective cohort study in the United States has observed that higher prepregnancy habitual intakes of folate are associated with a lower risk of GDM [11].

Similarly, the relationship between B_12_ and GDM is still conflicting [12]. Studies from India and UK indicated that maternal B_12_ insufficiency is associated with an increased risk of GDM [13,14]. In contrast, a recent prospective cohort study in China demonstrated that higher B_12_ in early pregnancy is associated with a higher risk of GDM [10]. Since these nutrients are interrelated in OCM, several studies have explored the combined effects of folate and B_12_ on GDM. Studies from Singapore and UK revealed that B_12_ insufficiency coupled with excess folate was associated with a higher risk of GDM [15,16]. Similar results were obtained in our previous study with a small sample size. In addition, we also found that the imbalance of serum folate and B_12_ (folate/B_12_) was more obviously related to GDM [17].

Although the relationship between Hcy and GDM remains controversial, a recent systematic review indicated that Hcy was significantly elevated among pregnant women with GDM compared with non-GDM women [18]. In addition to folate and B_12_, the blood concentration of Hcy is dependent on the activities of several B vitamin-dependent enzymes, such as *MTHFR*, *MTR*, and *MTRR* [19]. Inheritance of the specific genetic variants in the genes encoding these enzymes is considered the most vital determinant of OCM status in women of childbearing age [20]. Some common polymorphisms (*MTHFR*, rs1801133 and rs1801131; *MTR*, rs1805087; *MTRR*, rs1801394) may influence folate, vitamin B_12_, and Hcy levels [21,22,23]. Although limited studies have focused on the relationship between *MTHFR* rs1801133 single nucleotide polymorphisms (SNPs) and GDM [24,25], the associations between other OCM SNPs and GDM have not been well studied.

In light of the above findings, the relationship between OCM related nutrients and GDM is still controversial. SNPs in genes encoding *MTHFR*, *MTRR*, and *MTR* play an essential role in the OCM pathway. However, their role in GDM development is unclear. In addition, few studies have been performed to assess the combined effects of folate, B_12_, Hcy, and OCM related genetic polymorphisms on GDM. In this study, we aimed to jointly investigate maternal folate, B_12_, Hcy, and SNPs in the OCM pathway to test the following hypotheses: (1) higher maternal folate and lower B_12_ is associated with increased risk of GDM; (2) SNPs in OCM pathway is associated with GDM and (3) genetic variants in the OCM pathway modify the association between OCM nutrients and GDM.

## 2. Materials and Methods

### 2.1. Study Population

Pregnant women in this research were enrolled in the Gene-Environment-Nutrient-Epigenetics interaction on Maternal and Children health study (GENEMaC) between 2017 and 2018 in Tianjin, China. This cohort was established primarily to investigate gene-environment interactions of maternal arsenic exposure, arsenic metabolism-related nutrients, and gene polymorphisms on offspring’s health via epigenetic changes [26]. The research proposal was approved by the Ethics Committee of Tianjin Xiqing Hospital. All participants provided written informed consent before participating in this study. 

**Figure 1:**
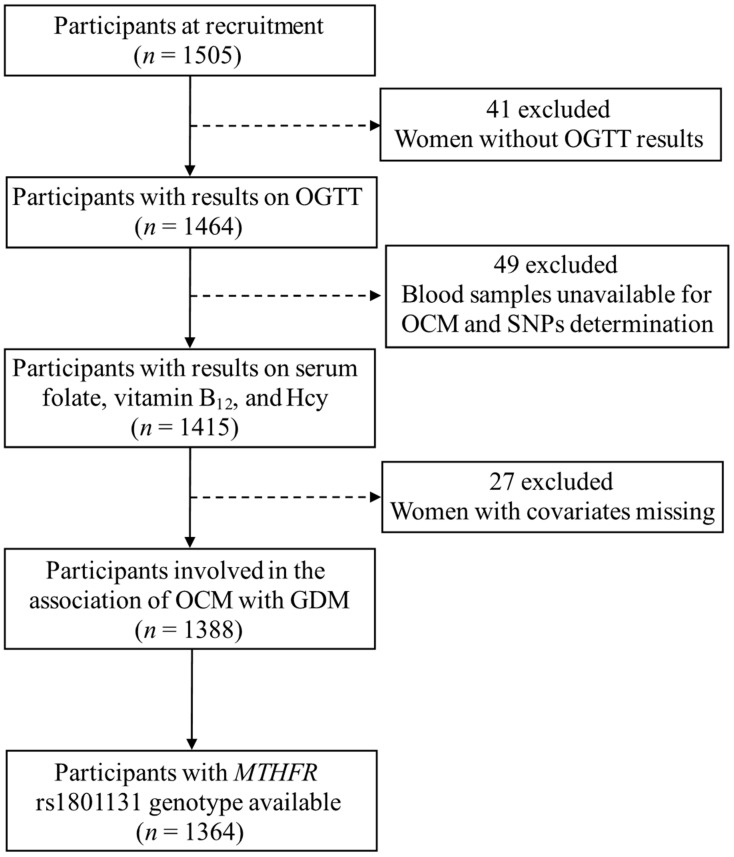
Study flowchart. GDM, gestational diabetes mellitus; Hcy, homocysteine; *MTHFR*, methylenetetrahydrofolate reductase; OGTT, oral glucose tolerance test; OCM, one-carbon metabolism; SNPs, single nucleotide polymorphisms.

A total of 1505 pregnancies who attended GDM screening at the Maternal and Child Health Care Hospital of Beichen District during 24–28 gestational weeks were enrolled in this cohort. The inclusion criteria were: (1) age ≥ 18 years, (2) residents of Tianjin with ≥one year of residence, and (3) intent to inhabit Tianjin in the next six years. The exclusion criteria were: (1) prepregnancy diabetes and previous GDM, (2) unable or unwilling to give informed consent or communicate with study staff. Of the 1505 participants, 1464 pregnant women completed the 75-g oral glucose tolerance test (OGTT). We excluded 49 pregnant women who did not have enough blood samples for OCM nutrients and SNPs determination, and 27 pregnant women with covariates missing, resulting in 1388 pregnancies included in the analysis of the association between OCM and GDM. In addition, we analyzed data from 1364 participants with *MTHFR* rs1801131 genotype data available for gene-nutrient interaction study (Figure 1). It should be noted that the GDM screening strategy in this area (Beichen District, Tianjin) is divided into two steps. Firstly, fasting plasma glucose (FPG) was used to rule out GDM (FPG < 4.4 mmol/L) and rule in GDM (FPG ≥ 5.1 mmol/L) in the community health centers (primary care providers) during 24-28 gestational weeks. Secondly, for pregnancies with FPG between ≥4.4 and <5.1 mmol/L, GDM diagnosis was performed by the OGTT examination at the Maternal and Child Health Care Hospital of Beichen District (secondary care provider). Therefore, the prevalence of GDM was higher in the present study.

### 2.2. Sample Collection and Covariates Assessment

The fasting blood sample was collected from each pregnancy during the GDM screening. Aliquots of blood sample and serum were obtained and then transferred to Tianjin Medical University for storage in freezers at −80 °C until analysis.

Baseline characteristics concerning an individual’s age, ethnicity, education, smoking and drinking habits, height, current and prepregnancy weight, parity, and family history of diabetes were obtained by a structured questionnaire via well-trained interviewers. Ethnicity was defined as Han nationality or Minority nationality. Education level was categorized according to the duration of education. Smoking and drinking were defined as never or ever before and during the pregnancy. Prepregnancy BMI (kg/m^2^) was estimated as prepregnancy weight (kg) divided by the square of height (m). 

### 2.3. Diagnosis of GDM

According to the diagnostic criteria recommended by the Ministry of Health of China, all the pregnancies underwent a GDM screening using a 75-g OGTT during 24–28 gestational weeks [27]. The Chinese diagnostic criteria agree with the International Association of Diabetes and Pregnancy Study Groups (IADPSG). Accordingly, a diagnosis of GDM can be made if one or more of the following glucose values are evaluated: fasting plasma glucose (FPG) ≥ 5.1 mmol/L, 1-h plasma glucose (1-h PG) ≥ 10.0 mmol/L, 2-h plasma glucose (2-h PG) ≥ 8.5 mmol/L.

### 2.4. Determination of OCM Related Nutrients

Determination of OCM nutrients was performed on maternal serum as previously described [26]. In brief, folate, and B_12_ concentrations were measured using an automated chemiluminescence immunoassay system (Architect-i2000SR Analyzer; Abbott Diagnostics, Chicago, USA). Hcy levels were determined using an automatic biochemical analyzer with an enzymatic cycling method (Dirui CS-T300; Dirui, Changchun, China).

### 2.5. Genotyping of OCM Related Genes

Functional polymorphisms in genes encoding main OCM related enzymes (Table S1) were genotyped according to the literature reports [20,21,22,23,28,29,30]: two SNPs in *MTHFR* (rs1801311 and rs1801133), three SNPs in *MTR* (rs1805087, rs2229276, and rs28372871), and seven SNPs in *MTRR* (rs1532268, rs162036, rs162048, rs16879334, rs1801394, rs326119, and rs3776455). Briefly, genomic DNA was extracted with RelaxGene Blood DNA System (Tiangen Biotech, Beijing, China) according to the manufacturer’s instructions. A high throughput-SNP (Hi-SNP) genotyping method based on three-round multiplex PCR coupled with next-generation sequencing was used to perform genotyping for the 12 SNPs (Biowing biotechnology, Shanghai, China) [31]. The overall genotyping success rate was >97%. For quality control, 10% of the samples were reanalyzed, and the concordance rate of the genotypes was >99%.

### 2.6. Statistical Analysis

The folate/B_12_ was calculated as folate divided by B_12_ according to our previous study [17]. Since the skewed distribution of folate, B_12_, Hcy, and folate/B_12_, these OCM indicators were reported as median (interquartile range, IQR). The baseline characteristics of the study participants were summarized using descriptive statistics (*n* [%] for categorical variables, median [IQR] for continuous variables). Wilcoxon Mann-Whitney *U* test was applied to determine the differences for continuous variables with skewed distribution. Chi-square test or Fisher’s exact test were used to examine the differences for categorical variables. Spearman correlation was performed to evaluate monotonic relationships between OCM indicators and glucose levels of the 75-g OGTT. OCM indicators, including folate, B_12_, Hcy, and folate/B_12_, were evaluated as continuous variables and categorical variables. Logistic regression was applied to calculate odds ratios (ORs) and 95% confidence intervals (CIs) for OCM indicators and SNPs on GDM. The restricted cubic spline (RCS) regression model with four knots was used to evaluate the potential nonlinear relationship among serum folate, B_12_, Hcy, folate/B_12_, and GDM risk. Potential maternal confounders, including age, ethnicity, education, drinking, smoking, parity, family history of diabetes, and prepregnancy BMI, were adjusted in all models. In addition, serum OCM indicators (folate, B_12_, and Hcy) were mutually adjusted in estimating the association of OCM indicators with GDM. When evaluating the relationship between different SNPs and GDM, both maternal characteristics and serum folate, B_12_, and Hcy were adjusted.

Since *MTHFR* rs1801311 was significantly associated with GDM under the dominant and additive models (see the Results section), associations between OCM indicators and GDM were reanalyzed under the stratification of rs1801311 genotypes. This allows us to target the subpopulation for intervention. Due to the small sample size of rs1801311 GG genotype, pregnant women were divided into GG/TG and TT groups according to the dominant model for further stratified analysis. Multiplicative and additive interactions were also performed to identify whether the effect of OCM indicators on GDM would be different in different genotype subgroups. A detailed description of these models could be found in our previous study [32]. Briefly, multiplicative interaction was assessed via the *p*-value (*p* _interaction_) of a cross-product interaction term of the OCM indicators and the rs1801311 genotype in a multiple logistic regression model. Additive interaction was evaluated through the relative excess risk due to interaction (RERI) using a multiple logistic regression model, and its 95% CI was computed with bootstrapping [33]. To estimate the overall association of the OCM nutrients with GDM stratified by rs1801311 genotype, the Bayesian kernel machine regression (BKMR) with 10,000 iterations was also employed. A more detailed description of this model can be found in our previous study [26]. Briefly, BKMR combines Bayesian and statistical learning methods to flexibly model the individual and joint effects of OCM mixtures on GDM using a Gaussian kernel function [34]. Results from these models could be used to (1) provide the exposure-response relationship for each OCM indicator on GDM when other indicators are fixed at their median; (2) evaluate the association of an IQR increase in a single OCM indicator on GDM when all the other indicators are fixed at either the 25th, 50th, or 75th percentile.

All statistical analyses were performed using R (version 4.0.2; R Project for Statistical Computing). BKMR was implemented with the R packages “bkmr” (version 0.2.0). A *p*-value < 0.05 was considered to be statistically significant.

## 3. Results

### 3.1. Baseline Characteristics

The demographic characteristics and OCM related nutrient concentrations of the study participants are shown in Table 1. Of the 1388 pregnancies, 274 (19.7%) were diagnosed with GDM. Pregnancies with GDM were more likely to be older and multiparous and have higher prepregnancy BMI and a family history of diabetes than non-GDM pregnancies. The median (IQR) levels of serum folate, B_12_, Hcy, and folate/B_12_ were 9.4 (6.2–14.6) ng/mL, 271 (214–337) pg/mL, 5.0 (4.5–6.0) μmol/L, and 35.1 (23.9–49.2), respectively. Compared with non-GDM women, subjects with GDM had significantly higher folate levels, lower B_12_ levels, and corresponding higher folate/B_12_. No significant differences were observed in Hcy levels between the two groups.

### 3.2. Associations between OCM Indicators and GDM

The correlations among serum folate, B_12_, Hcy, folate/B_12_, and plasma glucose levels are shown in Figure S1. Table 2 shows associations between OCM indicators and GDM. A significant dose-response relationship between serum folate and GDM risk was observed in crude and adjusted models (*p* for trend < 0.05). After adjustment for maternal age, ethnicity, education, drinking, smoking, parity, family history of diabetes, prepregnancy BMI, and serum B_12_ and Hcy, the association between folate and GDM was attenuated [OR (95% CI): Q1 = 1.0 (reference); Q2 = 1.47 (0.99, 2.26); Q3 = 1.61 (1.07, 2.49); Q4 = 2.28 (1.49, 3.61)]. In contrast to folate, higher serum B_12_ was negatively related to GDM after adjustment for multiple covariates [OR (95% CI): Q1 = 1.0 (reference); Q2 = 0.71 (0.50, 1.06); Q3 = 0.71 (0.49, 1.06); Q4 = 0.45 (0.30, 0.69)]. There was no significant association between serum Hcy and GDM occurrence. In line with the above findings, the odds ratios for GDM per IQR increase in folate, B_12_, and Hcy were 1.59 (95% CI: 1.22, 2.13), 0.76 (95% CI: 0.65, 0.92), and 1.04 (95% CI: 0.95, 1.15) after adjusting for multiple covariates, respectively. As shown in Table 2, higher folate/B_12_ was associated with increased ORs of GDM before and after adjustment for multiple covariates when treated as a categorical variable. The RCS regression model did not support the nonlinear relationship between serum folate and GDM. However, the RCS did identify a nonlinear association of serum B_12_ and folate/B_12_ with GDM (Figure S2).

### 3.3. Associations between OCM Related Gene Polymorphisms and GDM

The genotypes of the 12 SNPs were in Hardy-Weinberg equilibrium (*p* > 0.05). Among these SNPs in the OCM pathway, the genotypic distribution of the *MTHFR* rs1801131 SNP (TT, TG, and GG) was significantly different between the two groups (Table S1). Figure S3 shows the associations between OCM related SNPs and GDM under three genetic models. After adjustment for maternal characteristics and serum OCM indicators, *MTHFR* rs1801131 was associated with GDM in the dominant and additive models but not in the recessive model. Compared with pregnancies with TT genotype, pregnancies with TG (OR: 0.68; 95% CI: 0.49, 0.96) and GG (OR: 0.30; 95% CI: 0.07, 1.33) genotype had lower odds of GDM after adjustment for multiple covariates in the logistic regression analysis (Table 3). In the analysis of both mutant genotypes (GG/TG) under the dominant model, pregnancies with G alleles had a significantly lower risk of GDM than pregnancies with T alleles (OR: 0.65; 95% CI: 0.47, 0.91). However, pregnancies with GG genotype did not have a significantly lower risk of GDM than did pregnancies with TG/TT genotypes under the recessive model. In addition, an increased copy of the G allele was found to be associated with a lower risk of GDM (OR: 0.66; 95% CI: 0.48, 0.89) under the additive model (Table 3).

### 3.4. Combined Effects of OCM Indicators and rs18011311 Genotypes on GDM

The genotype-stratified analysis revealed that an IQR increase in maternal serum folate was associated with higher odds of GDM (OR: 1.66, 95% CI: 1.20, 2.30) among pregnancies with *MTHFR* rs1801131 TT genotype (Table 4). Similarly, the associations between serum B_12_ and GDM were more evident in pregnant women with *MTHFR* rs1801131 TT genotype (OR: 0.80, 95% CI: 0.65, 0.98) after adjustment of maternal characteristics and serum folate and Hcy concentrations. To further estimate whether the association between OCM indicators and GDM was modified by *MTHFR* rs1801131 genotypes, the interactions on the multiplicative and additive scales were evaluated. However, no significant interactions on the multiplicative and additive scales between OCM indicators and GDM were observed (Table 4).

**Figure 2:**
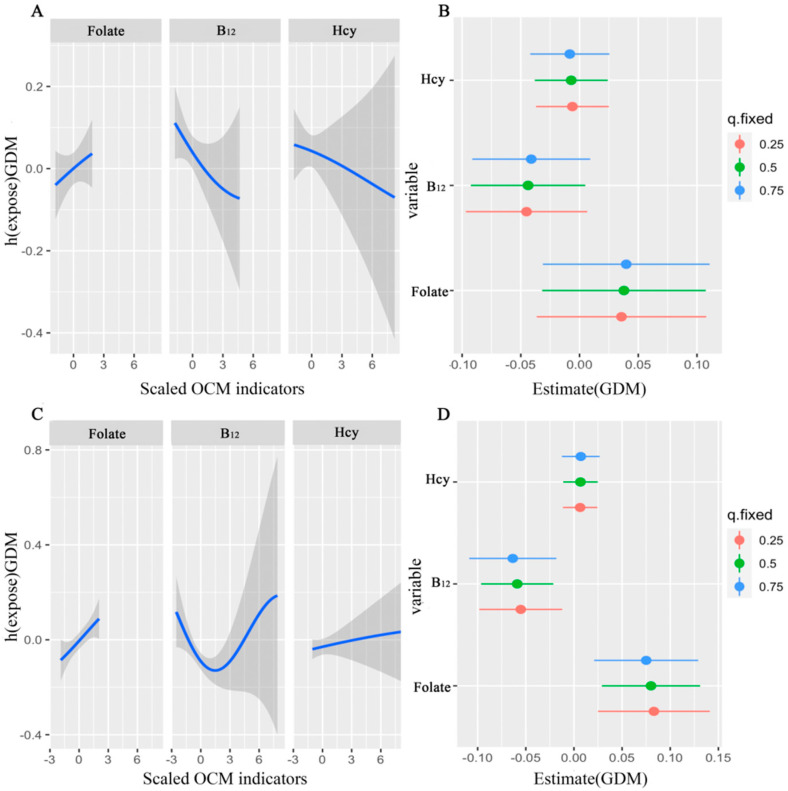
Joint effect of OCM indicator mixture on GDM estimated by Bayesian Kernel Machine Regression (BKMR). Model adjusted for age, ethnicity, education, drinking, smoking, parity, family history of diabetes, and prepregnancy BMI. (**A**,**C**) Univariate exposure-response functions and 95% confidence bands for each OCM indicator with the other two indicators fixed at their median among pregnancies with rs1801131 GG/TG and TT genotype, respectively. (**B**,**D**) Single nutrient association (estimates and 95% CI) between OCM indicator and GDM among pregnancies with rs1801131 GG/TG and TT genotype, respectively. This plot compares the GDM when a OCM nutrient is at the 75th vs. 25th percentile, when all the other OCM indicators are fixed at either the 25th, 50th, or 75th percentile.

The exposure-response functions of the three OCM indicators on GDM stratified by *MTHFR* rs1801131 genotypes are shown in Figure 2. Among pregnancies with rs1801131 GG/TG genotype, folate showed increasing association with GDM when B_12_ and Hcy were fixed at their median levels. However, B_12_ and Hcy displayed decreasing associations with GDM when the other two OCM indicators were fixed at their median levels, respectively (Figure 2A). Figure 2B showed the association of each OCM indicator with GDM when the single OCM indicator increased an IQR, where all of the other indicators are fixed at 25th, 50th, or 75th percentiles. However, there were no significant associations of folate, B_12_, and Hcy with GDM among pregnant women with rs1801131 GG/TG genotype. Among pregnancies with rs1801131 TT genotype, folate and Hcy showed increasing association with GDM. However, BKMR identified an individual U-shaped association between B_12_ concentrations and GDM (Figure 2C) while holding folate and Hcy at their median concentrations. As shown in Figure 2D, an IQR increase in folate was associated with a 0.07-unit (95% CI: 0.03, 0.12) increase in GDM risk when B_12_ and Hcy were fixed at their 50th percentile values (similar results were obtained when B_12_ and Hcy were fixed at their 25th and 75th percentile values). In contrast, an IQR increase in B_12_ was associated with a 0.07-unit (95% CI: −0.10, −0.03) decrease in GDM risk when folate and Hcy were fixed at their median values (similar results were obtained when folate and Hcy were fixed at their 25th and 75th percentile values). There was no significant association between Hcy and GDM when folate and B_12_ were fixed at different percentile values.

## 4. Discussion

In this gene-nutrient interaction study, we evaluated the combined effects of OCM related nutrients and gene polymorphisms on GDM in a Chinese pregnancy cohort for the first time. We found that serum folate concentrations were positive, whereas serum B_12_ concentrations were negatively associated with the risk of GDM. Notably, we found that the *MTHFR* rs1801131 TT genotype was significantly associated with an increased risk of GDM. Moreover, we found homozygous in pregnant women for the *MTHFR* rs1801131 TT genotype, higher folate, and lower B_12_ were more obviously associated with increased GDM risk.

Folic acid is widely used to prevent birth defects, with a recommended daily intake of 400 micrograms from prepregnancy until 12 weeks of pregnancy in many countries [35]. Emerging evidence suggests that periconceptional higher folate intake is associated with higher GDM risk [8,9]. However, inconsistent findings were found for prepregnancy habitual intakes of folate in the Nurse’s Health Study [11]. Although folic acid intake evaluated via the questionnaire may not accurately reflect folate levels in pregnant women, serum and red blood cell folate levels have also been associated with GDM [10,16]. Our preliminary study indicated that higher folate levels in mid-pregnancy can slightly increase maternal GDM risk [17]. In the present study, we expanded our findings with a large sample size. We found that serum folate levels are positively correlated with blood glucose levels and significantly associated with GDM risk in a dose-response manner. Our findings were consistent with recently published results [16,36], which indicated that higher maternal folate during pregnancy is associated with increased GDM risk. 

In contrast to folate, we found a significantly negative correlation between serum B_12_ levels and FPG. However, weak but not significantly positive correlations between B_12_, 1-h PG, and 2-h PG were observed in the present study (Figure S1). In addition, we found that the risk of GDM decreased with the increase of B_12_ levels. This, in turn, suggested that lower B_12_ was related to a higher risk of GDM (Table 2). Our findings were in line with the results of some previous studies, in which lower B_12_ was associated with a higher risk of GDM [13,14,15,16]. However, Chen et al. reported a positive relationship between B_12_ levels and GDM risk in a prospective study from Shanghai, China [10]. The reasons for the conflicting results are unclear. It was reported that the level of serum B_12_ decreased gradually with the progress of the pregnancy [37]. In our study, the median concentration of mid-pregnancy serum B_12_ was 271 pg/mL, which is lower than Chen’s report in early pregnancy (405.93 pg/mL). In addition, we found that the relationship between B_12_ and GDM was nonlinear (Figure S2B). When the serum B_12_ level reached about 400 pg/mL, the risk of GDM did not decrease with the increase of B_12_ levels. This may partly explain the differences between our study and Chen’s study. Since the proportion of pregnant women with serum B_12_ levels greater than 400 pg/mL (14.3%) was small, we did not observe a significantly positive association between B_12_ and GDM at higher serum B_12_ both in the logistic regression model and RCS model. Therefore, future studies should be performed in pregnancies with a wide range of serum B_12_ to investigate the dose-response association between B_12_ and GDM. 

Hcy is a surrogate marker for folate and B_12_ insufficiency. In this study, we confirmed that Hcy was negatively correlated with folate and B_12_. Although Hcy was negatively correlated with postprandial blood glucose, we did not find a significant association between Hcy and GDM in different statistical models (Table 2 and Figure S2C). Nevertheless, we discovered that folate/B_12_ is a sensitive index to evaluate the relationship between OCM nutrients and GDM. A higher folate/B_12_ value represents a relatively high folate level or a relatively low B_12_ level. In this study, we found that pregnancies with GDM have significantly higher folate/B_12_ values. Simultaneously, this index was positively associated with blood glucose levels and GDM risk. Although Chen’s study has shown that folate/B_12_ is not related to GDM [10], our findings suggested that the balance of folate and B_12_ may be necessary for the health of pregnant women. In addition, we also found that the relationship between folate/B_12_ and GDM was nonlinear (Figure S2D). This also partly explains the difference between our and Chen’s results in Shanghai, China [10]. 

Because of the involvement of *MTHFR*, *MTR*, and *MTRR* genes with the OCM pathway and the evidence that maternal folate and B_12_ imbalance during pregnancy increase GDM risk, we evaluated the influence of SNPs of these genes on the etiology of GDM in our study. The preliminary results showed that pregnancies with GDM were more prone to have a genotype TT for *MTHFR* rs1801131 (Table S1). After adjustment for multiple covariates, including maternal characteristics and OCM nutrients, *MTHFR* rs1801131 was found to be associated with GDM in the dominant model. Compared with the TT genotype, the GG/TG genotypes of rs1801131 were associated with a significantly lower risk of GDM before and after adjustment for multiple covariates (Table 3). Under the additive model, the presence of one or two copies of the G allele was associated with a reduced GDM risk. Our novel findings suggested that the minor G allele represents a protective factor in GDM. In turn, the TT genotype of *MTHFR* rs1801131 is a risk factor for GDM. Furthermore, we did not find a significant association between *MTHFR* rs1801133 and GDM, which agrees with the findings of previous studies [11,24,25]. 

In order to evaluate the effect modification of genetic variants on the associations of OCM indicators with GDM, a stratified analysis was performed. The results indicated that the associations between OCM indicators and GDM are heterogeneous in different genotypic groups. Pregnant women with TT genotype of *MTHFR* rs1801131 were more susceptible to folate and B_12_ related GDM (Table 4). These findings were also supported by the joint association of the OCM nutrients with GDM stratified by rs1801311 genotype using the BKMR model (Figure 2B,D), which showed significant associations of folate and B_12_ with GDM among pregnancies with rs1801131 TT genotype. By estimating the nonlinearity of the exposure-response function in the BKMR model, serum folate showed a positive association with GDM both in GG/TG and TT subgroups. However, the association between B_12_ and GDM was different between the two groups (Figure 2A,C). These findings also confirmed the RCS results (Figure S2A and Figure 2B), which showed a linear relationship between folate and GDM but a nonlinear relationship between B_12_ and GDM. 

The mechanisms of increased GDM risk with excess folate and low B_12_ are not well studied. Nutrients in the OCM pathway are interrelated, and disturbances in one nutrient will affect the status of others. The *MTHFR* rs1801131 G allele is associated with reduced enzyme activity. Therefore, pregnant women with the TT genotype can typically reduce 5, 10-methylene-THF to 5-methyl-THF. The reduction of 5, 10-methylene-THF by *MTHFR* is physiologically irreversible [38]. However, B_12_ deficiency will impede OCM by trapping folate as 5-methyl-THF since the *MTR*/*MTRR* mediates methyl group donation from 5-methyl-THF to Hcy requires B_12_ as a cofactor [39]. The deficiency of B_12_ will lead to an accumulation of 5-methyl-THF. This also explains why the relationship between folate, B_12_, and GDM is more dependent on the genotype of rs1801131 (Table 4 and Figure 2). More recently, an animal study indicated that dams that feed higher levels of 5-methyl-THF during pregnancy gained significantly more weight than dams that provide folic acid [40]. Their findings suggested that high-dose 5-methyl-THF exposure may play a role in the development of metabolic disease. On the other hand, OCM disturbance (enzyme activity or vitamins imbalance) substantially impacts the epigenome [41], such as DNA methylation, which may relate to GDM [42]. 

Our study has several limitations. First, we studied the relationship between OCM nutrients and GDM by expanding the sample size. However, the nature of the cross-sectional baseline study limited us to determining the causality of OCM nutrient imbalance and GDM. Nevertheless, the genetic models can be used to explore causal relationships among OCM related SNPs and GDM. Second, twelve SNPs were genotyped in the present study. Other SNPs related to the OCM pathway are encouraged to further explore the effect modification of genetic variants on the association of OCM nutrients with GDM. Third, total folate was determined in the present study, and we cannot distinguish between 5-methyl-THF and other forms of folate. Further confirmatory studies and mechanistic investigations are required to verify the potential role of 5-methyl-THF in GDM.

## 5. Conclusions

Our study demonstrated that higher folate and lower B_12_, as well as *MTHFR* rs1801131, may be independent risk factors for GDM. In addition, pregnancies with rs1801131 TT genotype are more susceptible to OCM related GDM. To our knowledge, this is the first epidemiologic study to use a gene-nutrient interaction approach to evaluate the combined effects of OCM related nutrients and gene polymorphisms on GDM risk. More importantly, our findings potentially lead to practically feasible GDM prevention via individualized intervention in the future.

## Figures and Tables

**Table 1:** Baseline characteristics of the study participants (*n* = 1388).

Characteristic		Overall (*n* = 1388)	Non-GDM (*n* = 1114)	GDM (*n* = 274)	*p*
Age (years)					
	<30	692 (49.9)	589 (85.1)	103 (14.9)	<0.001
	30–35	489 (35.2)	381 (77.9)	108 (20.1)	
	≥35	207 (14.9)	144 (69.6)	63 (30.4)	
Prepregnancy BMI (kg/m^2^)					
	<18.5	104 (7.5)	87 (83.7)	17 (16.3)	0.036
	18.5–24	771 (55.5)	636 (82.5)	135 (17.5)	
	24–28	375 (27.0)	287 (76.5)	88 (23.5)	
	≥28	138 (10.0)	104 (75.4)	34 (24.6)	
Ethnicity					
	Han nationality	1293 (93.2)	1031 (79.7)	262 (20.3)	0.095
	Minority nationality	95 (6.8)	83 (87.4)	12 (12.6)	
Education (years)					
	≤12	561 (40.4)	440 (78.4)	121 (21.6)	0.278
	12–15	436 (31.4)	351 (80.5)	85 (19.5)	
	>15	391 (28.2)	323 (82.6)	68 (17.4)	
Drinking					
	Never	1377 (99.2)	1106 (80.3)	271 (19.7)	0.462
	Ever	11 (0.8)	8 (72.7)	3 (27.3)	
Smoking					
	Never	1368 (98.6)	1101 (80.5)	267 (19.5)	0.092
	Ever	20 (1.4)	13 (65.0)	7 (35.0)	
Family history of diabetes					
	No	1260 (90.8)	1037 (82.3)	223 (17.7)	<0.001
	Yes	128 (9.2)	77 (60.2)	51 (39.8)	
Parity					
	Nulliparous	681 (49.1)	564 (82.8)	117 (17.2)	0.022
	Multiparous	707 (50.9)	550 (77.8)	157 (22.2)	
Folate (ng/mL)		9.4 (6.2–14.6)	9.1 (6.0–14.2)	10.5 (6.7–15.5)	0.009
B_12_ (pg/mL)		271 (214–337)	273 (218–344)	262 (198–317)	0.003
Hcy (μmol/L)		5.0 (4.5–6.0)	5.0 (4.5–6.0)	5.0 (4.4–6.0)	0.650
Folate/B_12_		35.1 (23.9–49.2)	34.1 (22.7–47.9)	40.6 (28.6–54.1)	<0.001

Values are *n* (%) or median (interquartile range). *p*-values were obtained by Chi-square test or Fisher’s exact test for categorical variables and Mann-Whitney *U* test for continuous variables.

**Table 2:** Odds ratios of OCM indicators for GDM (*n* = 1388).

OCM Indicators ^a^		Non-GDM	GDM	OR (95% CI) ^b^	*p*	*p* for Trend	OR (95% CI)^c^	*p*	*p* for Trend
Folate									
	Per IQR increase	1114	274	1.36 (1.07, 1.72)	0.011		1.59 (1.22, 2.13)	0.002	
	Q1 (<6.2)	288	52	1.0			1.0		
	Q2 (6.2–9.4)	286	67	1.30 (0.87, 1.93)	0.199		1.47 (0.99, 2.26)	0.076	
	Q3 (9.4–14.6)	275	71	1.43 (0.96, 2.12)	0.075		1.61 (1.07, 2.49)	0.033	
	Q4 (≥14.6)	265	84	1.76 (1.20, 2.58)	0.004	0.004	2.28 (1.49, 3.61)	<0.001	<0.001
B_12_									
	Per IQR increase	1114	274	0.83 (0.71, 0.97)	0.019		0.76 (0.65, 0.92)	0.004	
	Q1 (<214)	261	82	1.0			1.0		
	Q2 (214–271)	278	67	0.77 (0.53, 1.10)	0.154		0.71 (0.50, 1.06)	0.091	
	Q3 (271–337)	276	74	0.85 (0.60, 1.22)	0.384		0.71 (0.49, 1.06)	0.098	
	Q4 (≥337)	299	51	0.54 (0.37, 0.80)	0.002	0.006	0.45 (0.30, 0.69)	<0.001	<0.001
Hcy									
	Per IQR increase	1114	274	1.02 (0.93, 1.12)	0.656		1.04 (0.95, 1.15)	0.456	
	Q1 (<4.5)	267	77	1.0			1.0		
	Q2 (4.5–5.0)	283	65	0.80 (0.55, 1.15)	0.228		0.77 (0.54, 1.14)	0.195	
	Q3 (5.0–6.0)	282	65	0.80 (0.55, 1.16)	0.235		0.80 (0.56, 1.19)	0.268	
	Q4 (≥6.0)	282	67	0.82 (0.57, 1.19)	0.302	0.323	0.90 (0.62, 1.36)	0.631	0.650
Folate/B_12_									
	Per IQR increase	1114	274	1.02 (0.97, 1.07)	0.392		1.01 (0.96, 1.06)	0.683	
	Q1 (<23.9)	303	44	1.0			1.0		
	Q2 (23.9–35.1)	284	63	1.53 (1.01, 2.32)	0.047		1.58 (1.05, 2.44)	0.040	
	Q3 (35.1–49.2)	274	73	1.83 (1.22, 2.76)	0.004		1.94 (1.30, 2.98)	0.003	
	Q4 (≥49.2)	253	94	2.56 (1.72, 3.80)	<0.001	<0.001	2.56 (1.73, 3.91)	<0.001	<0.001

^a^ OCM indicators were evaluated as categorical variables (defined by quartiles) and continuous variables. ^b^ Crude model. ^c^ Adjusted for age, ethnicity, education, drinking, smoking, parity, family history of diabetes and prepregnancy BMI. In addition, in the folate group, the model was adjusted for serum B_12_ and Hcy concentrations; in the B_12_ group, the model was adjusted for serum folate and Hcy concentrations; in the Hcy group, the model was adjusted for serum folate and B_12_ concentrations; whereas in the folate/B_12_ group the model was adjusted for serum Hcy concentrations.

**Table 3:** Odds ratios of *MTHFR* rs1801131 genotype for GDM (*n* = 1364).

Genetic Variants		OR (95% CI) ^a^	*p*	OR (95% CI) ^b^	*p*
*MTHFR* rs1801131					
	TT	1.00		1.00	
	TG	0.67 (0.48, 0.92)	0.014	0.68 (0.49, 0.96)	0.027
	GG	0.36 (0.08, 1.57)	0.174	0.30 (0.07, 1.33)	0.113
	Dominant (GG/TG vs. TT)	0.65 (0.47, 0.89)	0.008	0.65 (0.47, 0.91)	0.012
	Recessive (GG vs. TG/TT)	0.40 (0.09, 1.72)	0.219	0.33 (0.07, 1.46)	0.142
	Additive (GG vs. TG vs. TT)	0.66 (0.49, 0.88)	0.006	0.66 (0.48, 0.89)	0.008

^a^ Crude model. ^b^ Adjusted for age, ethnicity, education, drinking, smoking, parity, family history of diabetes, prepregnancy BMI, and serum folate, B_12_ and Hcy concentrations.

**Table 4:** Odds ratios for GDM according to OCM indicators by *MTHFR* rs1801131 genotype (*n* = 1364).

OCM Indicators	GG/TG (*n* = 377) ^a^		TT (*n* = 987) ^a^		*p*_interaction_
	OR (95% CI)	*p*	OR (95% CI)	*p*	
Folate (Per IQR increase)	1.47 (0.74, 2.92)	0.274	1.66 (1.20, 2.30)	0.002	0.769
RERI (95% CI) ^a,b^	0.28 (−0.41, 1.05)				
B_12_ (Per IQR increase)	0.70 (0.47, 1.05)	0.081	0.80 (0.65, 0.98)	0.033	0.818
RERI (95% CI) ^a,b^	−0.05 (−1.23, 0.39)				
Hcy (Per IQR increase)	0.93 (0.69, 1.26)	0.653	1.05 (0.95, 1.17)	0.344	0.347
RERI (95% CI) ^a,b^	0.13 (−0.14, 0.38)				
Folate/B_12_ (Per IQR increase)	1.43 (0.91, 2.24)	0.118	1.00 (0.95, 1.06)	0.878	0.067
RERI (95% CI) ^a,b^	−0.46 (−1.06, 0.38)				

^a^ Adjusted for age, ethnicity, education, drinking, smoking, parity, family history of diabetes, and prepregnancy BMI. In addition, in the folate group, the model was adjusted for serum B_12_ and Hcy concentrations; in the B_12_ group, the model was adjusted for serum folate and Hcy concentrations; in the Hcy group, the model was adjusted for serum folate and B_12_ concentrations; whereas in the folate/B_12_ group the model was adjusted for serum Hcy concentrations. ^b^ The RERIs and its 95% CIs were calculated using per IQR increase in OCM indicators (folate, B_12_, Hcy, and folate/B_12_) and rs1801131 TT genotype.

## Data Availability

The datasets used and/or analysed during the current study are available from the corresponding author on reasonable request.

## Supplementary Materials

The following supporting information can be downloaded at: https://www.mdpi.com/article/10.3390/nu14061169/s1, Table S1: OCM related single nucleotide polymorphisms; Figure S1: Spearman’s correlation matrix of serum OCM indicators, and fasting, 1-h, and 2-h plasma glucose; Figure S2: Restricted cubic spline (RCS) regression analysis of OCM indicators with GDM risk; Figure S3: The associations between OCM related SNPs and GDM under three different genetic models: dominant, recessive, and additive.
