# Supplementary material for: Associations of Maternal rs1801131 Genotype in MTHFR and Serum Folate and Vitamin B12 with Gestational Diabetes Mellitus in Chinese Pregnant Women
Source: Nutrients. 2022 Mar 10;14(6):1169. doi: 10.3390/nu14061169 (PMC8954918; doi:10.3390/nu14061169)
Supplement: Supplementary file 1 [file nutrients-14-01169-s001.zip › nutrients-1584593-supplementary.pdf]

nutrients

MDPI

Article

Associations of Maternal rs1801131 Genotype in *MTHFR* and Serum Folate and Vitamin B_{12} with Gestational Diabetes Mellitus in Chinese Pregnant Women

Shuying Li [1,†], Xiubiao Tian [1,†], Yiyun Wang [2,3], Xumei Zhang [3,4], Liwen Zhang [2,3], Chen Li [2,3], Jing Li [5], Chunhua Wang [2,3], Huihuan Liu [6], Juan Liu [7], Hongjuan Liu [8], Xueli Yang [2,3], Weiqin Li [9], Junhong Leng [9], Xilin Yang [5], Naijun Tang [2,3] and Qiang Zhang [2,3,*]

1 Department of Endocrinology, Tianjin Xiqing Hospital, Tianjin 300380, China; lsy_tj2006@163.com (S.L.); 15022754681@163.com (X.T.)
2 Department of Occupational and Environmental Health, School of Public Health, Tianjin Medical University, Tianjin 300070, China; wyy2168390@163.com (Y.W.); zhangliwen@tmu.edu.cn (L.Z.); lichen@tmu.edu.cn (C.L.); wangchunhua@tmu.edu.cn (C.W.); yangxueli@tmu.edu.cn (X.Y.); tangnaijun@tmu.edu.cn (N.T.)
3 Tianjin Key Laboratory of Environment, Nutrition and Public Health, School of Public Health, Tianjin Medical University, Tianjin 300070, China; zhangxumei@tmu.edu.cn
4 Department of Nutrition and Food Science, School of Public Health, Tianjin Medical University, Tianjin 300070, China
5 Department of Epidemiology and Biostatistics, School of Public Health, Tianjin Medical University, Tianjin 300070, China; lijing_epi@tmu.edu.cn (J.L.); yangxilin@tmu.edu.cn (X.Y.)
6 Beichen District Women's and Children's Health Center, Tianjin 300499, China; 18522022122@163.com
7 Department of Biomedical Information and Library, Tianjin Medical University, Tianjin 300070, China; libliujuan@tmu.edu.cn
8 Department of Obstetrics and Gynecology, Tianjin Xiqing Hospital, Tianjin 300380, China; juanliuhong@163.com
9 Project Office, Tianjin Women and Children's Health Center, Tianjin 300070, China; liweiqin007@163.com (W.L.); ljhlzqljhlzq@163.com (J.L.)
* Correspondence: qiangzhang@tmu.edu.cn
† These authors contributed equally to this work.

Citation: Li, S.; Tian, X.; Wang, Y.; Zhang, X.; Zhang, L.; Li, C.; Li, J.; Wang, C.; Liu, H.; Liu, J.; et al. Associations of Maternal rs1801131 Genotype in *MTHFR* and Serum Folate and Vitamin B12 with Gestational Diabetes Mellitus in Chinese Pregnant Women. *Nutrients* **2022**, *14*, 1169. https://doi.org/10.3390/nu14061169

Academic Editor: A. Catharine Ross

Received: 20 January 2022
Accepted: 8 March 2022
Published: 10 March 2022

Publisher's Note: MDPI stays neutral with regard to jurisdictional claims in published maps and institutional affiliations.

Copyright: © 2022 by the authors. Submitted for possible open access publication under the terms and conditions of the Creative Commons Attribution (CC BY) license (https://creativecommons.org/licenses/by/4.0/).

Abstract: Circumstantial evidence links one-carbon metabolism (OCM) related nutrients, such as folate and vitamin B_{12}, with gestational diabetes mellitus (GDM). However, few studies have evaluated the combined effects of these nutrients with OCM related gene polymorphisms on GDM. This study investigated whether OCM related genetic variants modified the associations of folate and B_{12} with GDM. Logistic regression was used to estimate odds ratios (ORs) for OCM related nutrients and single nucleotide polymorphisms (SNPs) in genes encoding main OCM related enzymes (*MTHFR*, *MTR*, and *MTRR*) on GDM. Higher folate concentrations were associated with increased GDM risk (OR: 1.59; 95% CI: 1.22, 2.13). However, higher B_{12} concentrations were associated with reduced GDM risk (OR: 0.76; 95% CI: 0.65, 0.92). Pregnancies with *MTHFR* rs1801131 G alleles had a significantly lower risk of GDM than pregnancies with T alleles (OR: 0.65; 95% CI: 0.47, 0.91) under the dominant model. The genotype-stratified analysis revealed the association between folate and GDM (OR: 1.66, 95% CI: 1.20, 2.30) or B_{12} and GDM (OR: 0.80, 95% CI: 0.65, 0.98) was more evident in pregnancies with TT genotype. Higher folate and lower B_{12} are associated with GDM. Pregnancies with *MTHFR* rs1801131 TT genotype are more susceptible to OCM nutrient-related GDM.

Keywords: MTHFR; folate; vitamin B_{12}; one-carbon metabolism; gestational diabetes mellitus

Supplementary Materials:

Table S1. OCM related single nucleotide polymorphisms.

SNP	Gene	Genotype	*n* (%)	HWE *P*-value[a]	No-GDM	GDM	*P*-value[b]
rs1532268	*MTRR*	CC	1035 (74.6)	0.157	824 (79.6)	211 (20.4)	0.311
		CT	302 (21.7)		247 (81.8)	55 (18.2)	
		TT	29 (2.1)		26 (89.7)	3 (10.3)	
		Missing	22 (1.6)				
rs162036	*MTRR*	AA	935 (67.4)	0.842	738 (78.9)	197 (21.1)	0.147
		AG	399 (28.7)		332 (83.2)	67 (16.8)	
		GG	46 (3.3)		39 (84.8)	7 (15.2)	
		Missing	8 (0.6)				
rs162048	*MTRR*	AA	474 (34.2)	0.079	376 (79.3)	98 (20.7)	0.834
		AG	686 (49.4)		554 (80.8)	132 (19.2)	
		GG	203 (14.6)		163 (80.3)	40 (19.7)	
		Missing	25 (1.8)				
rs16879334	*MTRR*	CC	984 (70.9)	0.905	796 (80.9)	188 (19.1)	0.417
		CG	349 (25.1)		278 (79.7)	71 (20.3)	
		GG	32 (2.3)		23 (71.9)	9 (28.1)	
		Missing	23 (1.7)				
rs1801131	*MTHFR*	TT	987 (71.1)	0.166	774 (78.4)	213 (21.6)	0.021
		TG	355 (25.6)		300 (84.5)	55 (15.5)	
		GG	22 (1.6)		20 (90.9)	2 (9.1)	
		Missing	24 (1.7)				
rs1801133	*MTHFR*	AA	428 (30.8)	0.222	342 (79.9)	86 (20.1)	0.140
		AG	663 (47.8)		522 (78.7)	141 (21.3)	
		GG	275 (19.8)		232 (84.4)	43 (15.6)	
		Missing	22 (1.6)				
rs1801394	*MTRR*	AA	778 (56.1)	0.618	633 (81.4)	145 (18.6)	0.510
		AG	494 (35.6)		391 (79.1)	103 (20.9)	
		GG	85 (6.1)		66 (77.6)	19 (22.4)	
		Missing	31 (2.2)				
rs1805087	*MTR*	AA	1106 (79.7)	0.186	888 (80.3)	218 (19.7)	0.958
		AG	263 (18.9)		211 (80.2)	52 (19.8)	
		GG	9 (0.7)		7 (77.8)	2 (22.2)	
		Missing	10 (0.7)				
rs2229276	*MTR*	AA	407 (29.3)	0.147	332 (81.6)	75 (18.4)	0.187
		AG	671 (48.4)		526 (78.4)	145 (21.6)	
		GG	300 (21.6)		249 (83.0)	51 (17.0)	
		Missing	10 (0.7)				
rs28372871	*MTR*	TT	349 (25.1)	0.248	290 (83.1)	59 (16.9)	0.112
		TG	669 (48.2)		522 (78.0)	147 (22.0)	

		GG	330 (23.8)		270 (81.8)	60 (18.2)	
		Missing	40 (2.9)				
rs326119	*MTRR*	AA	438 (31.6)	0.157	343 (78.3)	95 (21.7)	0.294
		AC	685 (49.3)		561 (81.9)	124 (18.1)	
		CC	243 (17.5)		192 (79.0)	51 (21.0)	
		Missing	22 (1.6)				
rs3776455	*MTRR*	CC	674 (48.6)	0.313	548 (81.3)	126 (18.7)	0.673
		CT	562 (40.5)		446 (79.4)	116 (20.6)	
		TT	132 (9.5)		105 (79.5)	27 (20.5)	
		Missing	20 (1.4)				

Values are *n* (%).

[a] Hardy-Weinberg equilibrium checked in No-GDM subjects.

[b] P-values were obtained by Chi-square test or Fisher's exact test.

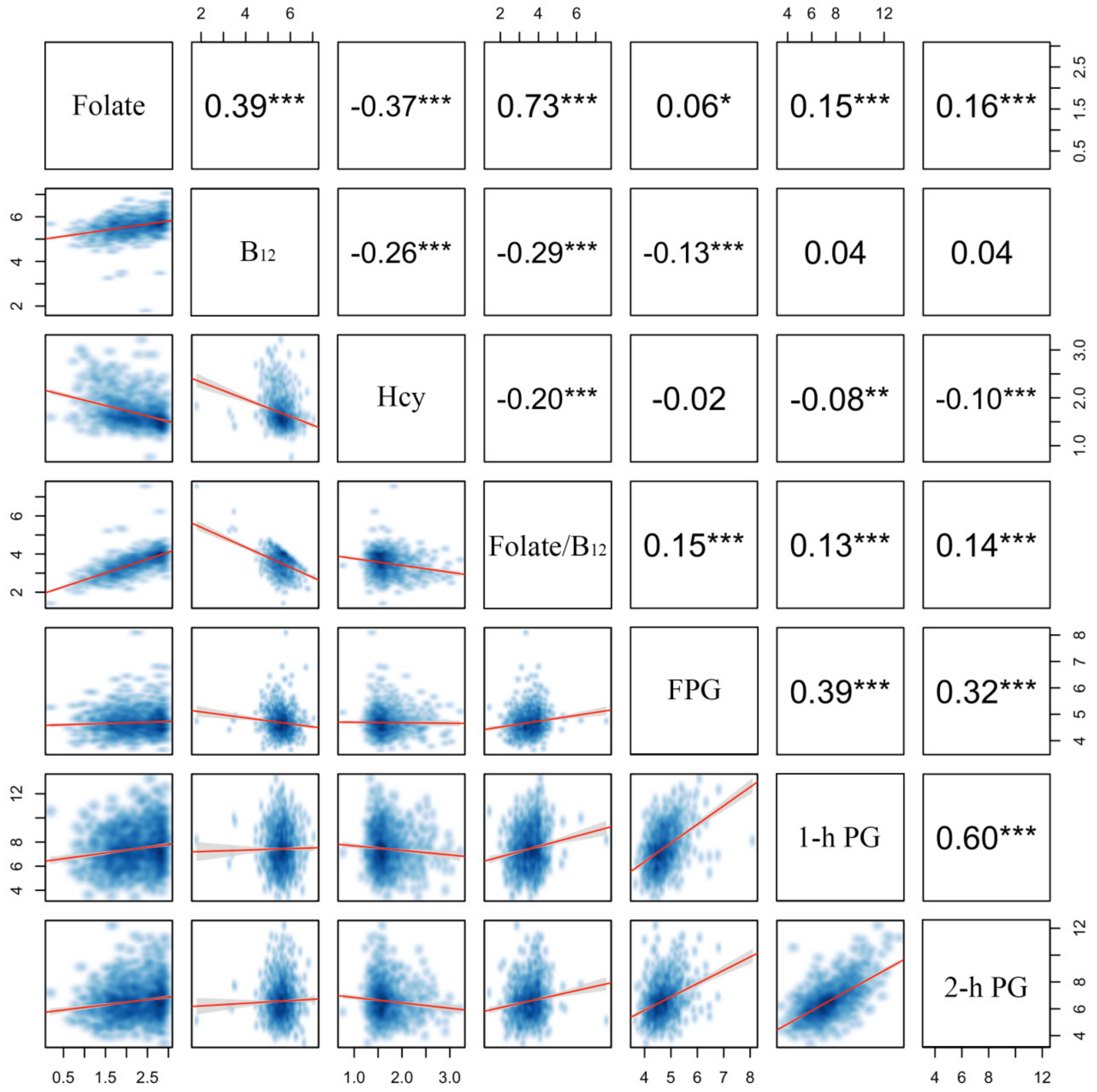

Figure S1. Spearman's correlation matrix of serum OCM indicators, and fasting, 1-h, and 2-h plasma glucose. * $P < 0.05$, ** $P < 0.01$, *** $P < 0.001$. FPG, fasting plasma glucose; B_{12}, vitamin B_{12}; Hcy, homocysteine; 1-h PG, 1-hour plasma glucose; 2-h PG, 2-hour plasma glucose.

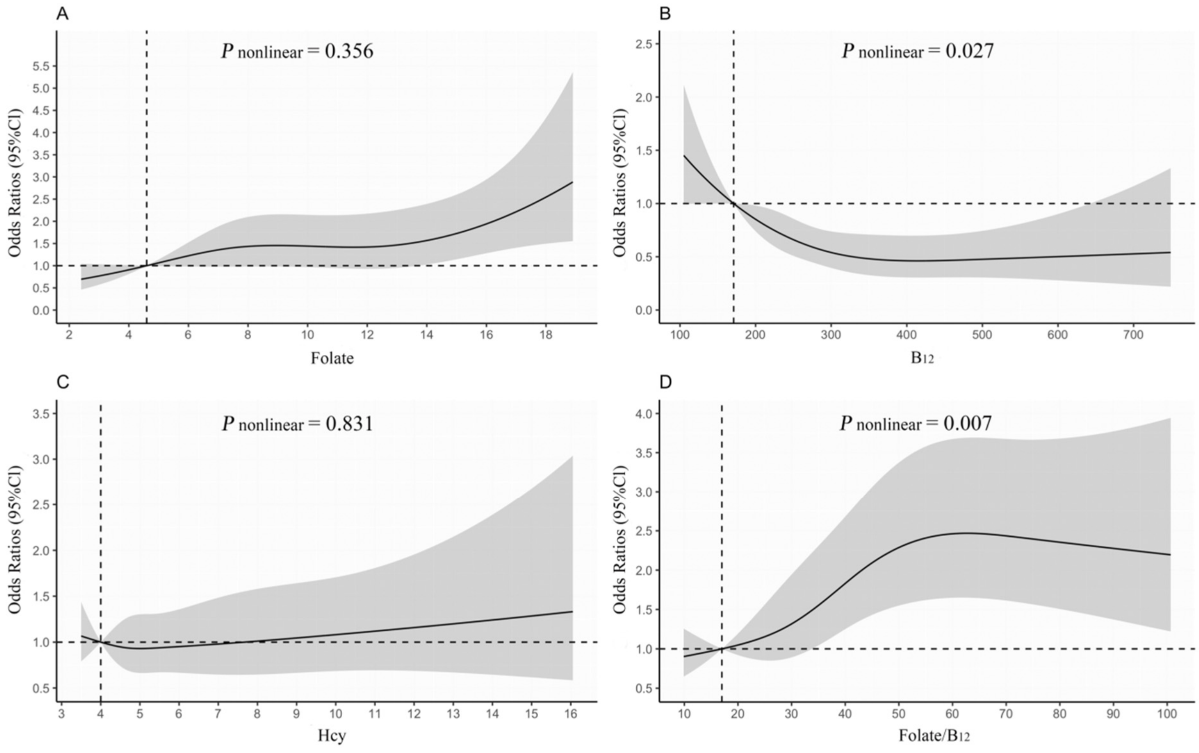

Figure S2. Restricted cubic spline (RCS) regression analysis of OCM indicators with GDM risk. Model adjusted for age, ethnicity, education, drinking, smoking, parity, family history of diabetes, and prepregnancy BMI. In addition, folate, B_{12}, and Hcy were mutually adjusted in estimating the association of another OCM indicator with GDM. 10% percentile value was selected as the reference level for each OCM indicator. (A) 4.6 ng/mL for serum folate; (B) 171 pg/mL for serum B_{12}; (C) 4.0 µmol/L for serum Hcy; (D) 17 for serum folate/B_{12}. The solid lines indicate eatimated ORs, and the gray areas represent 95% CI.

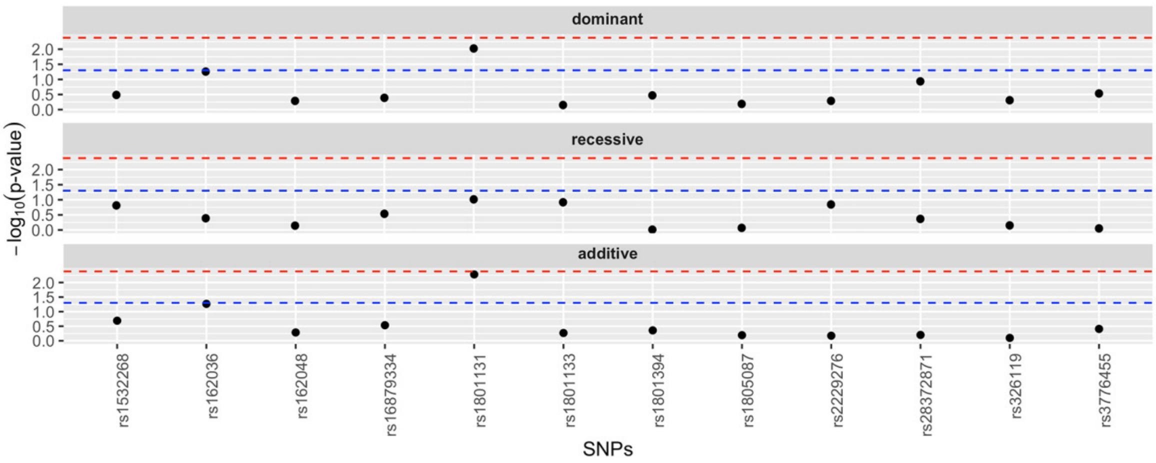

Figure S3. The associations between OCM related SNPs and GDM under three different genetic models: dominant, recessive, and additive. The -log *p* values from likelihood ratio test for each SNPs are showed for each genetic model. All models adjusted for age, ethnicity, education, drinking, smoking, parity, family history of diabetes, prepregnancy BMI, and serum folate, B_{12} and Hcy concentrations. The horizontal dotted lines indicate two different thresholds. One of them based on Bonferroni correction (red line), and another one in the nominal *P*-value which is set equal to 0.05 (blue line).
